# Extracorporeal techniques for the treatment of critically ill patients with sepsis beyond conventional blood purification therapy: the promises and the pitfalls

**DOI:** 10.1186/s13054-018-2181-z

**Published:** 2018-10-25

**Authors:** Ghada Ankawi, Mauro Neri, Jingxiao Zhang, Andrea Breglia, Zaccaria Ricci, Claudio Ronco

**Affiliations:** 1 Department of Internal Medicine and Nephrology, King Abdulaziz University, Jeddah, Saudi Arabia; 2 International Renal Research Institute of Vicenza (IRRIV), Vicenza, Italy; 3 Department of Nephrology, Dialysis and Transplantation, San Bortolo Hospital, Vicenza, Italy; 4 Department of Emergency and Critical Care Medicine, The Second Hospital of Jilin University, Changchun, China; 5 Department of Internal Medicine, University of Trieste, Trieste, Italy; 6 Department of Cardiology and Cardiac Surgery, Paediatric Cardiac Intensive Care Unit, Bambino Gesù Children’s Hospital, IRCCS, Rome, Italy

**Keywords:** Sepsis, Acute kidney injury, Renal replacement therapy, Extracorporeal technique, High volume hemofiltration, High cut-off membranes, Adsorption, Coupled plasma filtration adsorption

## Abstract

Sepsis is one of the leading causes of morbidity and mortality worldwide. It is characterized by a dysregulated immune response to infections that results in life-threatening organ dysfunction and even death. Bacterial cell wall components (endotoxin or lipopolysaccharide), known as pathogen-associated molecular patterns (PAMPs), as well as damage-associated molecular patterns (DAMPs) released by host injured cells, are well-recognized triggers resulting in the elevation of both pro-inflammatory and anti-inflammatory cytokines. Understanding this complex pathophysiology has led to the development of therapeutic strategies aimed at restoring a balanced immune response by eliminating/deactivating these inflammatory mediators. Different extracorporeal techniques have been studied in recent years in the hope of maximizing the effect of renal replacement therapy in modulating the exaggerated host inflammatory response, including the use of high volume hemofiltration (HVHF), high cut-off (HCO) membranes, adsorption alone, and coupled plasma filtration adsorption (CPFA). These strategies are not widely utilized in practice, depending on resources and local expertise. The literature examining their use in septic patients is growing, but the evidence to support their use at this stage is considered of low level. Our aim is to provide a comprehensive overview of the technical aspects, clinical applications, and associated side effects of these techniques.

## Background

The battle against sepsis is longstanding. Healthcare professionals continue to search for treatment modalities to improve the outcomes of patients suffering from this syndrome. The definition of sepsis has evolved over the years. An older definition was based on meeting two systemic inflammatory response syndrome (SIRS) criteria due to a presumed infection. In 2016, SIRS was replaced with the quick Sequential Organ Failure Assessment score (qSOFA), which consists of two of the following: increased breathing rate, change in level of consciousness, and low blood pressure. This was generated by national societies, including the Society of Critical Care Medicine (SCCM) and the European Society of Intensive Care Medicine (ESICM) [1, 2].

Sepsis is characterized by a dysregulated immune response to infections that results in life-threatening organ dysfunction. The exaggerated immune response beyond the infection site is multifactorial. Bacterial cell wall components (endotoxin or lipopolysaccharide (LPS)), known as pathogen-associated molecular patterns (PAMPs), and damage-associated molecular patterns (DAMPs) released by injured host cells play a major role in mounting this response with the subsequent release of both pro-inflammatory/anti-inflammatory cytokines. LPS has been found to cause a dose-dependent elevation in cytokines when injected systemically [3, 4]. Understanding of this complex mechanism has led to the development of treatment strategies aimed at restoring a balanced immune response by eliminating/deactivating these inflammatory mediators. Conventional therapy of sepsis typically starts with resuscitative measures; however, the only definitive therapy is adequate antibiotics and source control in surgical cases of sepsis [5]. Renal replacement therapy (RRT) is recommended in septic patients who develop acute kidney injury (AKI). Studies have shown no difference in outcomes comparing continuous RRT (CRRT) with intermittent RRT [6], and CRRT is generally reserved for hemodynamically unstable patients in need of fluid balance control [5]. Another application for RRT (hemofiltration in particular) is the extracorporeal removal of inflammatory mediators. In earlier studies, RRT alone was not sufficient to decrease serum cytokine levels [7], which led to the development of more effective extracorporeal techniques supported by controversial evidence at this stage. High volume hemofiltration (HVHF) or very high volume hemofiltration (VHVHF), high cut-off (HCO) membranes, adsorption alone, and coupled plasma filtration adsorption (CPFA) are among the major evolving strategies. These techniques are variably applied in different centers, depending on different clinician skills, equipment availability, and patient cases (i.e., surgical vs medical). In this review, we summarize the basic principles of these extracorporeal techniques. We also highlight the risks that should be carefully balanced against the potential benefits, given the low level of evidence supporting their effectiveness.

### High volume hemofiltration and very high volume hemofiltration

#### Terminology

**Fig. 1:**
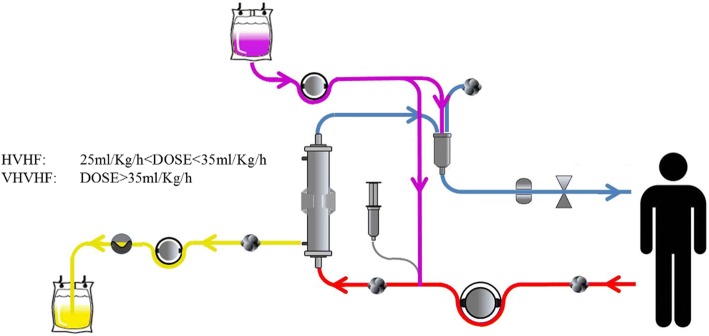
Circuit components in high volume hemofiltration (*HVHF*) and very high volume hemofiltration (*VHVHF*). Arterial line (*red*), ultrafiltrate (*yellow*), replacement fluid (*purple*), and venous line (*blue*)

The definition of HVHF remains controversial. Based on a consensus conference on the nomenclature of RRT held in Vicenza, Italy [8], HVHF was defined as continuous treatment with a convective target dose (prescribed) greater than 35 ml/kg/h. Doses greater than 45 ml/kg/h were defined as VHVHF. As a technique, it has been utilized for immunomodulation in the context of both AKI [9, 10] and sepsis. Our review will focus on the role of HVHF in sepsis. Although HVHF per se should be exclusively conducted by convective modalities, as this is the main mechanism by which inflammatory mediators are removed, some authors have delivered it combined with different approaches [11]. We will review HVHF and VHVHF simultaneously given the lack of standardized definitions, including the description of an additional complementary (diffusive) dose. HVHF and VHVHF circuits are shown in Fig. 1.

#### Technical aspects

HVHF and VHVHF are easily implemented in centers capable of performing conventional CRRT, as no additional component to the usual circuit is needed.

- Type of dialyzer: high flux dialyzer (Kuf > 25 ml/h/mmHg/m^2^).
- Blood flow rate (Qb): since HVHF and VHVHF require high ultrafiltration flows, required Qb typically must guarantee no excessive filtration fraction values (not > 25–30%).
- Dose: convective, greater than 35–45 ml/kg/h. The overall dose can be higher (50–70 ml/kg/h) due to a complementary diffusive component (continuous veno-venous hemodiafiltration (CVVHDF)).
- Replacement fluid (Qr): in pre- and/or post-dilution mode. For a given target dose, it is necessary to consider that the pre-dilution Qr must be higher than the post-dilution.
- Duration: tailored to the patient’s condition.
- Anticoagulation: heparin or citrate.

#### The evidence

The evidence for the effectiveness of high volume hemofiltration is presented in Table 1. The body of evidence is derived largely from small observational studies/randomized controlled trials (RCTs) [12–16]. The largest and only multi-center RCT to date is the IVOIRE trial [17]. It should be noted that different protocols were used with doses ranging from 40 to 70 ml/kg/h. A few studies have also utilized so-called pulse high volume hemofiltration (PHVHF), where intermittent very high-volume treatment is followed by conventional renal-dose hemofiltration [18–20]. Additionally, in a large Chinese cohort, higher doses of 50 ml/kg/h were compared to 85 ml/kg/h [21]. The results overall were inconsistent. Earlier observational studies have suggested a mortality benefit and improvement of hemodynamics, while later studies (IVOIRE trial in particular) did not confirm these findings. A recent systematic review [22] showed a pooled estimate of mortality risk ratio (RR) of 0.89 (95% CI 0.60–1.32; two trials; *N* = 156), and the strength of evidence was considered low. This finding was comparable to earlier meta-analyses by Clark et al. [23] and Lehner et al. [24]. The former showed the pooled odds ratio for 28-day mortality for HVHF compared with a standard volume hemofiltration (SVHF) of 0.76 (95% CI 0.45–1.29; *p* = 0.31), and the latter also demonstrated a lack of effect on mortality, with HFHV (OR 0.85; 95% CI 0.50–1.45; four trials; *N* = 473), PHVHF (OR 0.62; 95% CI 0.22–1.74; three trials; *N* = 85), or both combined (OR 0.85; 95% CI 0.60–1.22; seven trials; *N* = 558). It should be noted that some studies included by Lehner et al. [24] utilized hemodialysis and were not conducted exclusively in the context of sepsis. Furthermore, in a recent RCT involving a subpopulation of patients with septic shock secondary to burns, HVHF (versus standard therapy) was found to be effective in improving organ function but not survival [25]. To conclude, despite the promising results of earlier studies, HVHF seems to have no significant impact on short-term mortality, improvement in hemodynamics, or reduction in intensive care unit (ICU) or hospital length-of-stay (LOS).

**Table 1:** The main studies describing the effectiveness/limitations of high volume hemofiltration

	Honoré et al. 2000 [18]	Cole et al. 2001 [15]	Joannes-Boyau et al. 2004 [12]	Ratanarat et al. 2005 [19]	Cornejo et al. 2006 [14]	Piccinni et al. 2006 [13]	Boussekey et al. 2008 [16]	Joannes-Boyau et al. 2013 [17] (IVOIRE)
Study design	Cohort, uncontrolled prospective	Randomized crossover	Cohort, uncontrolled prospective	Cohort, uncontrolled prospective	Cohort, uncontrolled prospective	Retrospective	Prospective randomized study	Prospective, randomized, open, multicenter
Study population (n)	20 septic shock patients	11 septic shock patients	24 septic shock patients	15 severe sepsis patients	20 septic shock patients	80 septic shock patients	20 septic shock patients and AKI	140 septic shock patients and AKI
Prescribed dose	HVHF (4 h, 35 L of UF removed) followed by conventional CVVH for at least 4 days	8 h of HVHF (6 L/h) or 8 h of standard CVVH (1 L/h)	40–60 ml/kg/h for 96 h	HVHF 85 ml/kg/h for 6–8 h followed by CVVH 35 ml/kg/h for 16–18 h	100 ml/kg/h Single session of 12 h	HVHF (40 patients) at 45 ml/kg/h over 6 h followed by conventional CVVH compared to 40 historic patients treated with conventional therapy	HVHF 65 ml/(kg h) vs LVHF 35 ml/(kg h)	HVHF at 70 ml/kg/h vs SVHF at 35 ml/kg/h for 96 h
Survival/mortality	28-day observed survival of 45% compared to expected of 21% (*p* < 0.05)	Hospital mortality 54.5%	28-day mortality of 46% compared to predicted mortality of 70% (*p* < 0.075)	28-day mortality of 47% compared to predicted mortality of 68–72%	Observed hospital survival of 60% compared to expected survival of 37% (*p* < 0.03)	28-day survival of 55% compared to 27.5% in the conventional group (*p* < 0.05)	• ICU mortality of 33.3% in HVHF group vs 60% in LVHF group but not significantly different • 28-day mortality of 33.3% in the HVHF • group vs 50% in the LVHF group	• 28 day mortality of 37.9% in HVHF vs 40.8% in SVHF, (*p* = 0.94) • No difference in 60 and 90 days mortality
Length of ICU stay	–	–	–	–	–	Significant improvement (*p* < 0.002)	No difference	No difference
Hemodynamics	Improvement in 11/20 patients	Greater reduction in NE, HVHF vs standard CVVH (68% vs 7%; *p* = 0.02)	Significant improvement (*p* < 0.05)	Significant improvement (*p* = 0.001)	Improvement in 11/20 patients	Significant improvement (*p* < 0.05)	Improvement in VP dose in the treatment group (*p* = 0.004)	No difference
Safety	–	No AE	–	–	–	–	No AE	Hypokalemia (30% in HVHF vs 20% in SVHF (*p* = 0.1) Hypophosphatemia 88% in HVH vs 38 in SVHF (*p* = 0.01)

*HVHF* high volume hemofiltration, *LVHF* low volume hemofiltration, *SVHF* standard volume hemofiltration, *CVVH* continuous veno-venous hemofiltration, *UF* ultrafiltrate, *h* hour, *kg* kilogram, *NE* norepinephrine, *AE* adverse events, *VP* vasopressor

**Table:** 

HVHF is feasible and readily available in centers capable of performing conventional CRRT. The evidence to support its effectiveness in improving patient hemodynamics and mortality (although promising in earlier studies) is insufficient.	

### High cut-off membranes

#### Terminology

High cut-off (HCO) membranes are characterized by a large pore size (average pore diameter (20 nm) compared with the standard high-flux membrane (10 nm). Our focus will be the use of HCO membranes in CRRT in the context of sepsis.

**Fig. 2:**
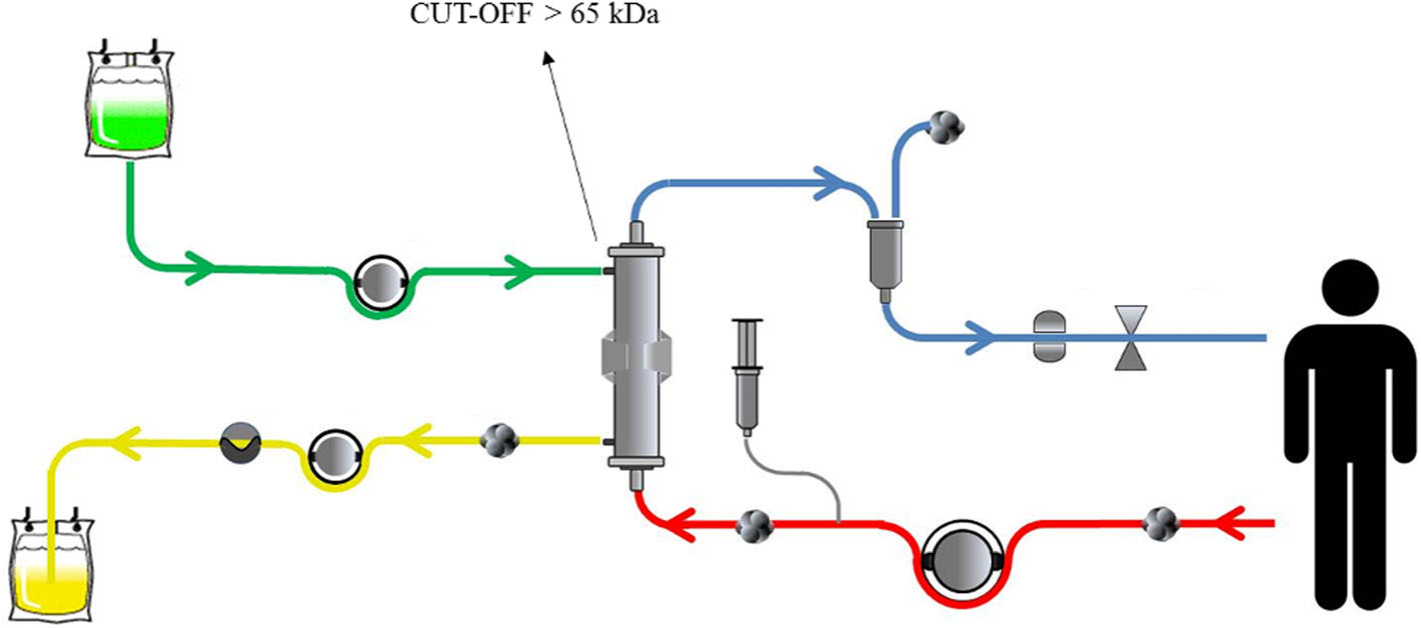
Circuit components using high cut-off membranes. Arterial line (*red*), ultrafiltrate (*yellow*), dialysate (*green*), and venous line (*blue*)

A HCO membrane circuit is shown in Fig. 2.

#### Technical aspects

HCO membrane use is similar to the standard RRT prescription, including the choice of anticoagulation. The prescribed dose is typically in the range from 25 to 40 ml/kg/h, as recommended by Kidney Disease Improving Global Outcomes (KDIGO) guidelines [26]. Convective modalities would maximize the HCO membrane ability to remove pro/anti-inflammatory mediators [27], but the excessive albumin loss commonly observed with the use of such membranes dictates the utilization of only diffusive modalities.

The use of HCO membranes is not limited to sepsis, which is the focus of our review. High-cut-off hemodialysis, for instance, is also used in the management of AKI in patients with rhabdomyolysis and multiple myeloma.

#### The evidence

The evidence for the effectiveness of HCO membranes is presented in Table 2. The body of evidence comes from small RCTs and observational studies [28–34]. Overall, the studies are suggestive of a decrease in inflammatory cytokines and improvement of hemodynamics, along with an improvement in ICU patient severity of illness scores as reported by Morgera et al. [27–30]. One study comparing continuous veno-venous hemodialysis with HCO membranes (HCO-CVVHD; at a dose of 35 ml/kg/h) vs CVVHDF (at a dose of 45 ml/kg/h) showed an ICU mortality benefit (37.5 and 87.5% for HCO-CVVHD and CVVHDF groups, respectively (*p* = 0.03)), but no significant difference was found in ICU-LOS and in-hospital mortality [32]. Furthermore, the High Cut-Off Sepsis study (HICOSS) comparing conventional and HCO membranes was stopped prematurely after enrolment of 81 patients due to a lack of 28-day mortality benefit (HCO (31%) vs conventional (33%)) and lack of a difference in vasopressor requirement or ICU-LOS [35].

**Table 2:** The main studies describing the effectiveness/limitations of high cut-off membranes

	Morgera et al. 2003 [28, 29]	Morgera et al. 2004 [27]	Morgera et al. 2006 [30]	Haase et al. 2007 [31]	Chelazzi et al. 2016 [32]	Kade et al. 2016 [34]	Villa et al. 2017 [33]
Study design	Prospective single-center pilot trial	Prospective RCT	Prospective RCT	Double-blind, crossover RCT	Retrospective, observational	Retrospective, single center	Observational prospective multicenter study
Study population (n)	16 septic shock patients	24 patients with sepsis-induced AKI	30 septic patients with AKI	10 septic patients with AKI	16 patients with Gram-negative sepsis	28 patients with septic shock	38 patients with septic shock and AKI
Prescribed dose	Intermittent HP-HF over 5 days for 12 h/day alternating with conventional HF (12 h)	CVVH (UF 1 L/h) vs CVVH (UF 2.5 L/h) vs CVVHD (dialysate flow rate of 1 L/h) vs CVVHD (dialysate flow rate of 2.5 L/h)	HCO vs conventional HF	4 h of HCO-IHD and 4 h of HF-IHD	HCO 35 ml/kg/h vs CVVHDF 45 ml/kg/h	HCO-CVVHDF	HCO-CVVHD for 72 h
Results	High IL-6 elimination	Increasing UF volume or dialysate flow led to a significant increase in IL-1ra and IL-6 clearance rates (*p* < 0.00001)	Significant reduction in VP dose in the HCO group (*p* = 0.0002) Clearance rates for IL-6 and IL-1ra were significantly higher in the HCO group (*p* < 0.0001)	Greater decrease in plasma IL-6 levels (*p* = 0.05), plasma IL-8 (*p* = 0.02) and plasma IL-10 (*p* = 0.04) in the HCO group	ICU mortality rates were 37.5 and 87.5% for HCO and HF groups, respectively (*p* = 0.03) ICU LOS: 16 and 9 days (HCO- and HF-group; *p* = 0.03). Improvement of hemodynamics in the HCO group (*p* < 0.03)	Significant reduction in IL-10 and IL-12 levels	Significant reduction in circulating levels of TNFα and IL-6 among survivors
Safety or S/E	High cumulative 12-h protein loss (7.60 g; IQR 6.2–12.0)	High protein and albumin losses with 2.5-L/h HF mode	None	Albumin loss of 7.7 g in the HCO group vs < 1.0 g (*p* < 0.01)	–	–	–

*RCT* randomized controlled trial, *AKI* acute kidney injury, *HCO* high cut-off, *CVVH* continuous veno-venous hemofiltration, *CVVHD* continuous veno-venous hemodialysis, *CVVHDF* continuous veno-venous hemodiafiltration, *HP-HF* high permeability hemofiltration. *UF* ultrafiltration, *HCO-IHD* high cut-off intermittent hemodialysis, *HF-IHD* high flux intermittent hemodialysis, *HF* hemofiltration, *TNF* tumor necrosis factor, *IL* interleukin, *LOS* length of stay, *VP* vasopressors, *IQR* interquartile range

**Table:** 

Based on the reviewed literature, there is no evidence to support the use of HCO hemofiltration in sepsis. Lack of standardized definitions of dialysis membranes [36] has contributed to the paucity of high-quality data supporting their use.	

### Adsorption

#### Terminology

Adsorption is performed in the form of hemoperfusion (HP), plasma perfusion, or coupled plasma filtration adsorption (CPFA) (detailed in the “Coupled plasma filtration adsorption (CPFA)” section). HP involves passage of blood through a hemofilter where mediators are adsorbed to the membrane surface or through a sorbent-containing cartridge. We will focus on the use of adsorption in sepsis; however, it has also been studied in the context of cardiopulmonary bypass surgery and other conditions. In sepsis, it is generally advocated for treating patients with suspected Gram-negative sepsis or septic shock.

**Fig. 3:**
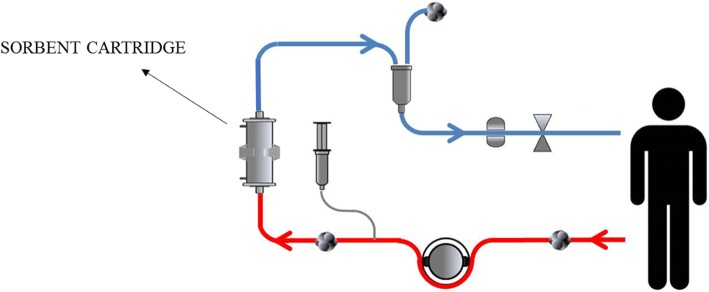
Circuit components in adsorption. Arterial line (*red*) and venous line (*blue*)

Circuit components in adsorption are shown in Fig. 3.

#### Technical aspects

Adsorption can be used in isolation or in combination with HD or continuous veno-venous hemofiltration (CVVH). More than one session is often required to overcome the possible rebound. Prescription (including the duration of therapy) depends on the adsorption cartridge used (summarized in Table 3) and should always be guided by the user manual. This is a rapidly evolving area and we will focus on the widely used cartridges.

**Table 3:** The commonly used adsorption cartridges and their prescriptions

	Toraymyxin	Cytosorb	Oxiris	LPS adsorber	HA 330
Composition	Polymyxin B-immobilized fiber blood-purification column	Porous polymer beads	AN69-based membrane, surface treated with PEI and grafted with heparin	Synthetic polypeptide bound to porous polyethylene discs	Styrene divinylbenzene copolymers
Indication	Severe sepsis and septic shock	Severe sepsis and septic shock Cardiac surgery with SIRS	Severe sepsis and septic shock	Severe sepsis and septic shock	Severe sepsis and septic shock
Toxins removed	Endotoxins	Cytokines/chemokines Anaphylatoxins Myoglobin Free hemoglobin Bilirubin/bile acids Toxins/metals Drugs	Endotoxin Cytokines	Endotoxins	Cytokines Complements Free hemoglobin
Prescription	2-h session daily for 2 consecutive days	Up to 24-h therapy daily for 2–7 consecutive days	Prescribed dose > 35 ml/kg/h (60% convective). Filter replacement after 24 h or if there is no reduction in VP dose by 50%. Treatment should be stopped if VP are reduced by > 50% or after 3 days of treatment in case of no-response	2–6 h. One session is usually sufficient to achieve improvement. Repeated procedures can be performed	2–6 h daily for 2 days
Blood flow rate (ml/min)	80–120	150–700	100–450	150 ± 50	100–300
Anticoagulation	Heparin	Heparin or citrate	Heparin	Heparin	Heparin or citrate
Additional features	Polymyxin B antimicrobial effect	Largest surface area	Lower risk of thrombogenicity by adsorbing antithrombin-III from the blood		

*CRRT* continuous renal replacement therapy, *LPS* lipopolysaccharides, *PEI* polyethyleneimine, *SIRS* systemic inflammatory response syndrome, *VP* vasopressors

#### The evidence

The evidence for adsorption is presented in Tables 4, 5, 6, and 7).

**Table 4:** The main studies describing the effectiveness/limitations of the polymyxin B-immobilized fiber column

	European pilot study (2005) [37]	EUPHAS (2009) [38]	Japan Registry (2014) [41]	ABDO-MIX (2015) [39]	Japan Registry (2016) [40]	EUPHAS 2 (2016) [42]
Study design	Multicenter, open-label, pilot, RCT	Multicenter, open-label, prospective RCT	Propensity-matched analysis	Multicenter, prospective RCT	Propensity-matched analysis	Retrospective study
Study population (n)	36 patients with intra-abdominal sepsis	64 patients with intra-abdominal sepsis or septic shock	PMX = 642 intra-abdominal sepsis patients vs 590 propensity score-matched pairs	232 patients with intra-abdominal septic shock/peritonitis	Septic shock patients with CRRT-requiring AKI	357 patients with suspected Gram-negative sepsis
EAA assessment	Measured	Not measured	Not measured	Not measured	Not measured	Some centers
Prescribed dose	1 session (2 h)	2 sessions (2 h)	1–2 sessions	1–2 sessions (2 h)	1–2 sessions	1–2 sessions (2 h)
Timing (h)	24–48 (from diagnosis)	24 (from abdominal surgery)	24 (from surgery)	12 (from surgery)	24 (from starting CRRT)	24–48 (from diagnosis)
Survival/ mortality	Mortality, 29% in the PMX group vs 28% in the control group (*p* = 0.749)	• PMX group had a significant reduction in 28-day mortality (adjusted HR 0.36; 95% CI 0.16–0.80; *p* = 0.01) • PMX group had a significant reduction in hospital mortality rate (adjusted HR 0.43; 95% CI 0.21–0.90; *p* = 0.026)	28-day mortality was 17.1% in the treatment group and 16.3% in the control group (*p* = 0.696)	• 28-day mortality 27.7% in the treatment group vs 19.5% in the control group (*p* = 0.14) • 90-day mortality was 33.6% in the treatment group vs 24% in the control group (*p* = 0.10)	• The 28-day mortality was 40.2% in the treatment group and 46.8% in the control group (*p* = 0.003) • 28-day mortality in patients receiving 2 PMX was 35.7% vs 42.6% in the group treated with one session	• 28-day survival 54.5% • ICU survival 55.2% • Hospital survival 50% • Patients with abdominal sepsis treated within 24 h survival 64.5%
Length of ICU stay	13.2 ± 9.4 days in the PMX; vs 17.0 ± 9.4 days	No significant difference	–	No significant difference	–	–
Hemodynamics	Significant improvement in the PMX group	Significant reduction in VP dose in the treatment group	–	No significant difference	No significant difference	–
Other results	No significant difference in the change of IL-6 levels compared to baseline	–	–	–	–	–
Safety	Higher AE (mainly change in vitals in the treatment arm)	No adverse events reported	–	6 severe adverse events (hemorrhagic episodes in the treatment group) Platelet drop	–	Significant platelet drop with no clinical implications

*CRRT* continuous renal replacement therapy, *EAA* endotoxin activity assay, *PMX* polymyxin, *AE* adverse event, *VP* vasopressors

**Table 5:** The main studies describing the effectiveness/limitations of the Cytosorb cartridge

	Schädler et al. 2013 [51]	Friesecke et al. 2017 [50]	Schädler et al. 2017 [52]	Kogelmann et al. 2017 [49]
Study design	Multicenter, open label, RCT	Prospective interventional single center	Multicenter, open label, RCT	Case series
Study population (n)	43 septic patients with ALI	25 septic shock patients	97 septic patients with ALI or ARDS	16 septic shock patients
IL-6 assessment (pg/ml)	–	> 1000	Average of 565	–
Prescribed dose	ST vs ST + HP (6 h/day for 7 days)	One session in the pre-filter mode. Further treatments at the discretion of the study physicians	HP vs no HP (6 h/day for up to 7 days) RRT as clinically indicated in both groups	HP in the pre-filter mode (1–5 treatments)
Timing	–	Within 24 h	–	< 24 to > 48 h (outcomes better in the early group)
Survival	28-day mortality 28% in the treatment group vs 24% in the controls (*p* = 0.84) 60-day mortality (39% in the treatment group vs 32% the controls (*p* = 0.75)	–	28-day mortality 36.2% in the treatment group vs 18.0% in the controls (*p* = 0.073) 60-day mortality of 44.7% in the treatment group vs 26.0% in the controls (*p* = 0.039)	The actual 28-day, ICU, and hospital mortality was 61.54%, 73.08%, and 80.77%, respectively, compared with 89.9% as predicted by APACHE II score
Hemodynamics	–	Significant reduction in VP requirements compared to baseline	–	Significant reduction in VP requirements compared with baseline
Other results	Significant reduction in IL-6	Significant reduction in IL-6	IL-6 reduction in the HP group compared with no HP	–
Safety	Modest reduction in platelet count (< 10%) and albumin (< 5%)	No AE	1 drop in platelets in the treatment group	No AE

*RCT* randomized controlled trial, *ALI* acute lung injury, *ARDS* acute respiratory distress syndrome, *IL* interleukin, *ICU* intensive care unit, *AE* adverse event, *ST* standard therapy, *HP* hemoperfusion, *RRT* renal replacement therapy, *APACHE II* Acute Physiology and Chronic Health Evaluation II, *VP* vasopressor

**Table 6:** The main studies describing the effectiveness/limitations of the HA 330 cartridge

	Huang et al. 2010 [56]	Huang et al. 2013 [57]
Study design	RCT	RCT
Study population (n)	44 sepsis or septic shock patients	46 ALI/extra-pulmonary sepsis patients
EAA assessment	–	–
Prescribed dose	HP for 2 h for 3 days	HP for 2 h for 3 days
Survival	• ICU mortality 12.5% in HA vs 45.0% in the controls (*p* = 0.02) • Hospital mortality 37.5% in HA vs 50.0% in the controls (*p* = 0.81) • 28-day mortality 45.8% in HA vs 55.0% in controls (*p* = 0.47)	• ICU mortality 24% in HA vs 57.14% in the controls (*p* = 0.02) • 28-day mortality 28% in HA vs 66.7% in the controls (*p* = 0.009)
Length of ICU stay (days)	12.4 ± 3.1 in HA vs 19.5 ± 4.0 in controls (*p* = 0.03)	15.5 ± 4.0 in HA vs 19.4 ± 3.1 in controls (*p* = 0.04)
Hemodynamics	Significant reduction in VP dose in the HA group vs increase in the control group (*p* = 0.01)	Significant reduction in VP dose in the HA group vs increase in the control group (*p* = 0.032)
Other results	Significant difference in IL-8 and IL-6 levels between the two groups at day 3 (*p* = 0.03 and 0.01, respectively)	Significant difference in IL-1 and TNF-a in BAL fluid between the two groups (*p* = 0.02 and 0.04, respectively)
Safety	• 1 patient with fever in the HA group • Transient reduction in platelet counts in the HP group	–

*RCT* randomized controlled trial, *ALI* acute lung injury, *EAA* endotoxin activity assay, *HA* hemadsorption, *HP* hemoperfusion, *ICU* intensive care unit, *TNF* tumor necrosis factor, *BAL* broncho-alveolar lavage, *VP* vasopressor, *IL* interleukin

**Table 7:** The main studies describing the effectiveness/limitations of LPS adsorbers

	Yaroustovsky et al. 2009 [60]	Ala-Kokko et al. 2011 [61]	Adamik et al. 2015 [62]
Study design	Observational	Case series with matched controls	Observational
Study population (n)	13 Gram-negative sepsis	24 septic shock patients and endotoxaemia.	62 septic shock and suspected Gram-negative
EAA assessment	–	More than 0.3 considered endotoxaemia	EA [0.70 EA units (0.66–0.77)].
Prescribed dose	Two sessions with a maximum duration of 120 min/patient Alteco adsorber (*n* = 6) and toraymyxin (*n* = 7)	2-h LPS HP	LPS elimination + ST vs ST 1–2 sessions
Timing	–	Within 36 h	Within 24 h
Survival	–	–	No effect
Length of ICU stay	–	–	No effect
Hemodynamics	Improved MAP	Decreased VP	Significant improvement in the treatment group
Other results	Decrease in endotoxin and procalcitonin levels	Decreased endotoxin levels	Decreased endotoxin levels
Safety		Low platelets, two patients requiring transfusion but no bleeding	

*EAA* endotoxin activity assay, *ST* standard therapy, *MAP* mean arterial pressure, *VP* vasopressors, *LPS* lipopolysaccharide, *HP* hemoperfusion

##### Polymyxin B-immobilized fiber column

The body of evidence for the polymyxin B-immobilized fiber column (PMX; Toraymyxin) comes from three major RCTs [37–39], data from two registries [40–42], and four meta-analyses that included the earlier smaller RCTs conducted in Japan [43–46]. To date, the evidence remains largely mixed. Data from the EUPHAS trial [38] suggest a mortality benefit (28-day mortality, 32% in the treatment group vs 53% in the control group; adjusted HR 0.36, 95% CI 0.16–0.80) and a hemodynamic benefit, but no effect on ICU-LOS (20.3 days in PMX group (95% CI 15.0–25.5 days) vs 18.3 days (95% CI 8.8–27.8 days) in the control group; *p* = 0.72). In contrast, data from the ABDO-MIX trial [39] suggest no mortality benefit (28-day mortality 27.7% in the treatment group vs 19.5% in the control group; *p* = 0.14; OR 1.5872; 95% CI 0.8583–2.935) and no impact on hemodynamics or ICU-LOS (11 days in the PMX-HP vs 10 days in the control group; *p* = 0.49). However, cartridge clotting and treatment failure rates were high in this trial (two PMX sessions were completed in only 69.8% of patients), which may partially explain the findings. Similarly, two retrospective studies reported by Iwagami et al. [40, 41] showed conflicting results. The first showed no significant difference in 28-day mortality (17.1% in the treatment group compared with 16.3% in the control group; *p* = 0.696). In contrast, the second study showed 28-day mortality benefit (40.2% in the PMX group vs 46.8% in the control group; *p* = 0.003). A recent meta-analysis [47] (seven RCTs, 841 patients) suggested a reduction of mortality (RR 0.65; 95% CI 0.47–0.89; *p* = 0.007), which was similar to the results of the previous meta-analyses conducted by Cruz et al. [44] (RR 0.50; 95% CI 0.37–0.68), Qiu et al. [45] (RR 0.24; 95% CI 0.16–0.38), and Zhou et al. [46] (RR 0.57; 95% CI 0.45–0.72). However, it was considered to be low-quality evidence due to the serious risk of bias. All the trials were small, open-label, and conducted in single centers in Japan. Additionally, most of the trials involved a surgical population, and thus the results may need to be interpreted with caution when applying the results to medical ICU patients.

The EUPHRATES trial conducted in North America (NCT01046669) has recently completed enrolment, and the results will soon be officially available. Preliminary reports, however, suggest that less than a 5% mortality difference was recognized in the “per protocol population” (*N* = 244, 31.9 vs 36.9%) and that the decrease was not statistically significant [48]. Further analysis of potential benefits in subgroups of patients is ongoing. Of note, PMX use was found to be largely safe. Thrombocytopenia and leukopenia are common but generally not clinically significant.

##### Cytosorb

The evidence for cytosorb (CS) is limited to case reports/series and a few RCTs, but it is growing. Observational data [49, 50] suggest improvement in hemodynamics and a trend towards decreased mortality. In addition, a reduction of interleukin (IL)-6 levels has been observed, consistent with the findings of Kellum et al. examining the effect of CS on IL-6/other cytokines in brain-dead potential donors [47]. Two RCTs [51, 52] have also shown a reduction of IL-6 levels, but this result was not associated with an improvement in mortality, although the studies were not powered to evaluate mortality, and in the latter study [52] the treatment group had more severe disease compared with the controls. A clinical registry on the use of CS involving 22 countries has been developed, and according to its most recent report, the use of CS in 135 septic patients was not associated with side effects. The observed mortality was 65% compared with a predicted risk of death of 78% based on the Acute Physiology and Chronic Health Evaluation II (APACHE II) score. A marked reduction of IL-6 levels was also observed [53]. One drawback is that CS does not capture endotoxins and IL-10. Albeit in an in vitro study testing the removal of a broad spectrum of toxic PAMPS and DAMPS [54], except for the tumor necrosis factor (TNF)-α trimer, hemadsorption using CS reduced the levels of a broad spectrum of cytokines, DAMPS, PAMPS, and mycotoxins by > 50%. Because of the unspecific mediator-elimination properties of CS, it has been widely studied in the context of cardiac surgery. In a recent report by Bernardi et al. [55], CS was applied to elective cardiopulmonary bypass surgery patients. This was not associated with reduction in IL-6 level or improved clinical outcomes. This finding may partially be explained by the finding that cytokine levels in this patient population were not as high as in septic patients. Another caveat is the treatment duration (average 191 ± 56 min compared with treatment lasting for up to 7 days in other studies). This emphasizes the effect of both the initial level of cytokines and the treatment frequency/duration on the extent of cytokine reduction.

##### HA-330

In the context of sepsis, two small RCTs described effectiveness of HA-330 in decreasing inflammatory mediators, along with an improvement in hemodynamics, mortality, and ICU-LOS [56, 57]. One of the trials was conducted in septic patients with acute lung injury, in which there was marked improvement in respiratory parameters in the HP group [57].

##### Modified AN69 (Oxiris)

The evidence supporting the use of modified AN69 is limited to case series [58, 59]. In the study reported by Shum et al. [59], oXiris®-CVVH was delivered to six patients with septic AKI, and the results were compared to 24 severity-matched historical controls undergoing standard therapy. The results demonstrated that the SOFA score was reduced after 48-h CVVH from the value at ICU admission by 38% in the oXiris® group, while it was increased by 3% in the control group. No significant difference was observed in ICU and in-hospital mortality between the two groups. More studies investigating its effectiveness are ongoing (NCT01948778, NCT02600312).

##### LPS adsorbers (Alteco)

Evidence for LPS adsorbers was obtained from case reports/series [60–62] that showed a decrease in endotoxin level as well as improvement in patient hemodynamics with no significant side effects. A feasibility study (The ASSET trial) was unfortunately terminated early due to difficulty recruiting patients (NCT02335723).

**Table:** 

Hemoperfusion is a well-tolerated and feasible technique. There is no robust evidence for the use of HP in sepsis; however, some studies suggest a trend toward hemodynamic improvement and decreased mortality with its use.	

### Coupled plasma filtration adsorption

#### Terminology

Coupled plasma filtration adsorption (CPFA™) is a combination of separation of plasma from the cellular components of blood with a highly permeable filter, followed by sorbent adsorption of the plasma component with a styrene resin to remove a number of different cytokines and then reinfusion of the purified plasma before the hemofilter to finally simultaneously provide CRRT for renal/fluid support. The advantage of CPFA is the lack of direct contact between blood cells with the sorbent material, which leads to improved biocompatibility.

**Fig. 4:**
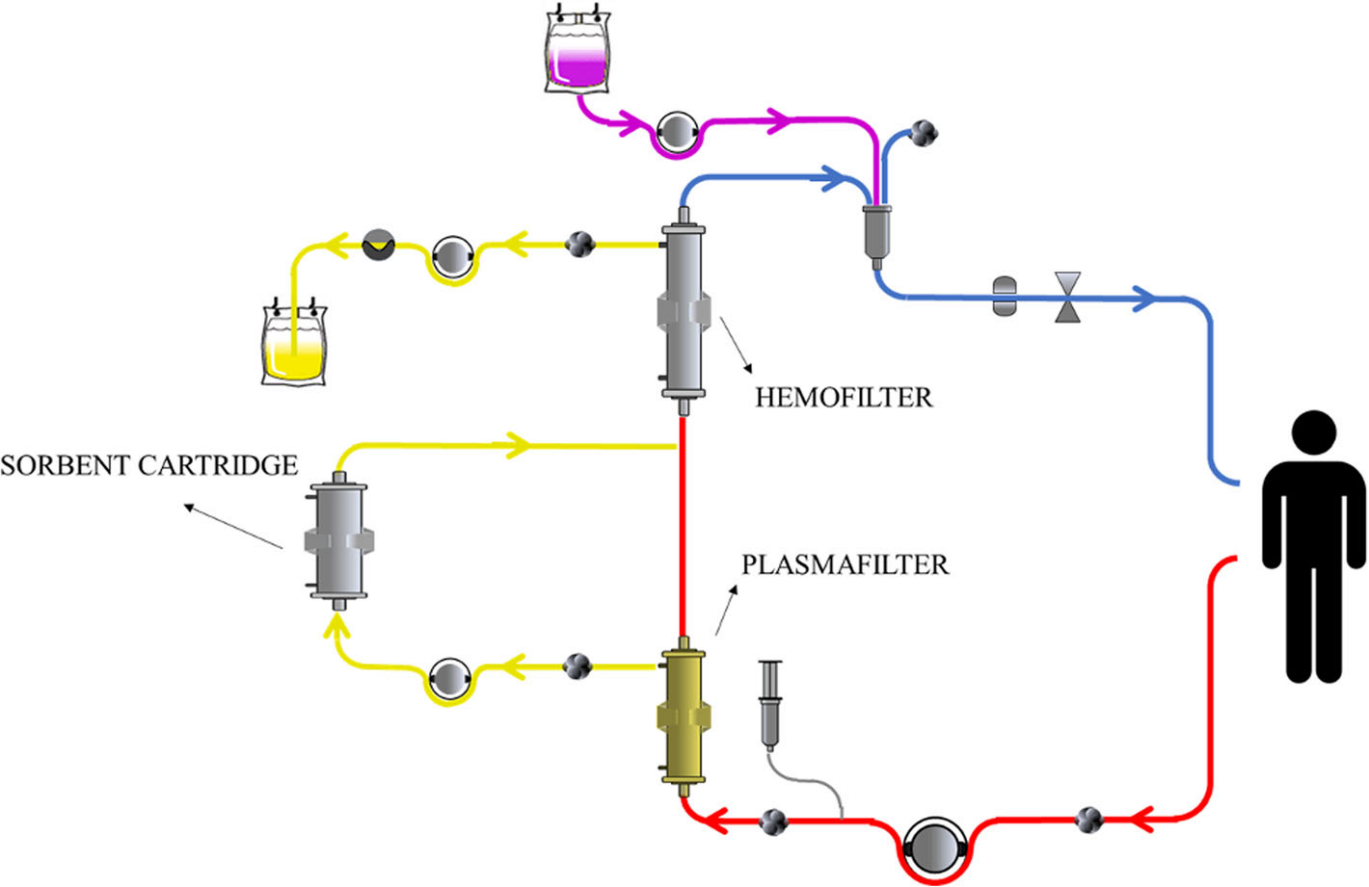
Circuit components in coupled plasma filtration adsorption (CPFA). Arterial line (*red*), plasma (*yellow*, pre-hemofilter), ultrafiltrate (*yellow*, post-hemofilter), replacement fluid (*purple*), and venous line (*blue*)

A CPFA circuit is shown in Fig. 4.

#### Technical aspects

- Blood flow rate (Qb): typically, 150–220 ml/min (max 250 ml/min).
- Plasma flow rate: 17–20% of the blood flow rate (35–40 ml/min).
- Ultrafiltration rate: max 2500 ml/h (equivalent to 35 ml/kg/h in a 70 kg patient).
- Replacement fluid (Qr): usually in post-dilution mode.
- Duration: daily for five days lasting for at least 10 h/day.
- Anticoagulation: the typical anticoagulant used is heparin, but citrate has been used safely [63] and may represent an attractive alternative given the high rate of clotting with CPFA.

The effectiveness of CPFA is dose-dependent, and volumes of plasma cleared in excess of 0.18 L/kg/day are typically associated with better outcomes [64].

#### The evidence

The evidence for the use of CPFA is largely derived from small, observational studies [65–68], which suggested no benefit in terms of survival or ICU-LOS but potential improvement in hemodynamics, interestingly, in a dose-dependent fashion. A recent meta-analysis that involved 14 studies suggested a potential improvement in survival, but the studies were not high quality and had a small size [69]. The largest RCT to date is the COMPACT trial [64] involving 192 patients randomized to either standard of care versus CPFA plus standard of care. There was no difference in hospital mortality (controls (47.3%) vs CPFA (45.1%); *p* = 0.76) or ICU-free days during the first 30 days (6.8 vs 7.5; *p* = 0.35). The trial was stopped prematurely due to futility. COMPACT I [64] highlighted a few technical issues related to CPFA. First, nearly half of the patients in the CPFA arm did not reach the planned dose. Clotting of the circuit was the cause in 48% of cases despite the use of heparin. Second, cost was a major concern. The high cost contributed to under-treatment, as replacing the circuit when the treatment was interrupted due to clotting was not possible. Finally, CPFA requires adequate staff training because it is somewhat complex. Two other large RCTs are ongoing (COMPACT II (NCT01639664) and ROMPA (NCT02357433)), which should help expand the body of evidence concerning the feasibility and effectiveness of CPFA.

**Table:** 

CPFA is feasible but evidence supporting its effectiveness to date is limited. Furthermore, it is expensive, labor-intensive, and associated with multiple technical issues that often lead to under-treatment. Well organized staff training programs are required when considering the utilization of this technique.	

## Adverse events (the pitfalls)

It is important to recognize that the above-described techniques are not without side effects. Adverse events associated with their use should not be overlooked, but rather carefully monitored. In particular, in light of the current low level of evidence supporting the effectiveness of these techniques, their use should be highly individualized and only practiced in centers with adequate experience and capabilities for vigilant monitoring of patients. Common to all the techniques are extracorporeal circuit-related adverse events. The risks of bleeding, clotting/changes in anti-coagulation requirements, drops in platelets counts, and catheter complications are all well-known. Also common to all techniques is the important issue of drug removal. Antimicrobials, in particular, are of utmost importance [70–72]. In septic patients, antibiotics are the only proven therapy. The risk of antibiotic removal, or under-dosing of patients, should be carefully accounted for by accurate drug level monitoring. In an in vitro study examining the effect of adsorption (as an example) on drug removal [73], vancomycin, for instance, showed a significant reduction in levels at different time points following adsorption therapy. Additional antibiotics doses may often be required. An unpredictable loss of beneficial molecules such as albumin (particularly in the case of HCO membrane use), other nutrients, and amino acids is another important consideration. Electrolyte imbalances (hypophosphatemia and hypokalemia in particular) are other important issues that could be harmful, particularly in critically ill patients, and should be carefully monitored. The risk is more pronounced in HVHF use, as emphasized by Clark et al. [23]. Frequent monitoring and pre-prepared protocols for electrolyte replacement are important. Furthermore, all these techniques add some complexity to the usual RRT prescription. Adequate personnel training is required before these techniques can be applied. Finally, all these techniques share the disadvantage of high cost. The current level of evidence is not in favor of utilizing these techniques given the high cost. As far as technique-specific adverse events, adsorption is associated with thrombocytopenia and leukopenia (as highlighted in the above-described studies). HVHF places patients at risk of hemodynamic instability and hypothermia with high convection [74]. HVHF also results in an increased nursing workload (more frequent procedures, such as bag changes in particular), which can lead to the possible introduction of error. With regard to HCO use, albumin loss is particularly significant in comparison to other techniques. This can be minimized by applying CVVHD, as convective clearance is more associated with albumin loss compared with diffusive clearance, while increasing the effluent flow to increase cytokine clearance [27].

**Table:** 

Adverse events, such as exposure to extracorporeal circuit, antibiotic removal, loss of beneficial molecules, electrolyte imbalances, increased cost, and increased work load, should be carefully monitored.	

## Future directions

The high morbidity and mortality associated with sepsis, along with the magnitude of health care resources utilized when managing septic patients, explain the ongoing efforts to optimize therapy. Therapeutic strategies aiming at elimination of the inflammatory mediators involved in the pathogenesis of sepsis represent an attractive and evolving area. As summarized in our review, different extracorporeal techniques have been studied, and the body of evidence to support their use is growing but remains controversial at this stage. With the current level of evidence, these techniques should not be widely adopted until the level of evidence to support their use is more robust.

In this section, we share our personal view on the use of extracorporeal therapies in sepsis. Across the different modalities, there has been a trend towards hemodynamic improvement. Its effect, however, in terms of decreasing mortality and length of ICU/hospital stay is, for the most part, limited and somewhat conflicting. Nevertheless, it is important to note that although mortality and length of ICU/hospital stay are important outcomes, they may not be the only desired outcomes in this setting. These techniques may potentially serve as a bridge to stabilize critically ill patients until more definitive therapies take place.

The application of these extracorporeal techniques is generally highly variable worldwide depending on resources and local expertise. Therapy should be tailored to the individual patient condition. Furthermore, side effects should be carefully monitored. In our opinion, the stage at which the patient is captured may influence the choice of modality. Earlier in the course, when levels of endotoxins and cytokines are extremely high, the application of adsorption/CPFA may help deactivate and decrease the peak elevation of these mediators, resulting in clinical outcomes. Later in the course, adsorption techniques may not be as effective, as the damage caused by the elevated mediators has already taken place, and utilization of the potential benefits of HCO membranes or HVHF for organ support may be more appropriate. Further studies are needed to confirm the theoretical effect of timing of the start of therapy on the utilized modality.

Another area in which future trials are still needed is adsorption. To date, the evidence to support its effectiveness is limited; however, in our opinion, there are important considerations before concluding that it is ineffective. Adsorption in particular seems to be dependent on the initial level (the higher the initial level of the desired solute for clearance, the more effective is the therapy, which may translate to clinical outcomes). This phenomenon has been demonstrated in the EUPRATES trial (NCT01046669), as benefit was observed in a subpopulation of patients with higher endotoxin levels. Treatment frequency also seems to have an effect (the more frequent the therapy, the more effective it becomes). This phenomenon has been demonstrated in studies utilizing adsorption in the treatment of poisoning, but the concept applies in other acute conditions [75]. Moreover, the range of molecular weights removed by adsorption is wider (in comparison to other techniques). Therefore, if applied early in the course during which the inflammatory mediator level is at its peak and continued sufficiently long, adsorption may represent a more promising tool in comparison to the other techniques. However, this hypothesis remains to be confirmed.

## Conclusions

To date, evidence is insufficient to support the use of extracorporeal techniques in sepsis. However, further efforts to try to identify research gaps in an attempt to optimize their use in septic patients are warranted. Our review aims to provide a comprehensive overview concerning both the benefits and risks of these techniques. Further studies to guide clinicians in the application of these techniques in the proper clinical setting are still needed.

## Abbreviations

AKI: Acute kidney injury
APACHE II: Acute Physiology and Chronic Health Evaluation II
CI: Confidence interval
CPFA: Coupled plasma filtration adsorption
CRRT: Continuous renal replacement therapy
CS: Cytosorb
CVVH: Continuous veno-venous hemofiltration
CVVHDF: Continuous veno-venous hemodiafiltration
DAMPs: Damage-associated molecular patterns
ESICM: European Society of Intensive Care Medicine
HCO: High cut-off
HCO-CVVHD: High cut-off continuous veno-venous hemodialysis
HCO-HD: High-cut-off hemodialysis
HR: Hazard ratio
HVHF: High volume hemofiltration
ICU: Intensive care unit
IL: Interleukin
KDIGO: Kidney Disease Improving Global Outcomes
Kuf: Ultrafiltration coefficient of the dialyzer
LOS: Length of stay
LPS: Lipopolysaccharide
OR: Odds ratio
PAMPs: Pathogen-associated molecular patterns
PHVHF: Pulse high volume hemofiltration
PMX: Polymyxin B-immobilized fiber column
Qb: Blood flow rate
Qr: Replacement fluid
qSOFA: Quick sequential organ failure assessment score
RCT: Randomized controlled trial
RR: Risk ratio
RRT: Renal replacement therapy
SCCM: Society of Critical Care Medicine
SIRS: Systemic inflammatory response syndrome
SVHF: Standard volume hemofiltration
TNF: Tumour necrosis factor
VHVHF: Very high volume hemofiltration
